# Conservation and Role of Electrostatics in Thymidylate Synthase

**DOI:** 10.1038/srep17356

**Published:** 2015-11-27

**Authors:** Divita Garg, Stephane Skouloubris, Julien Briffotaux, Hannu Myllykallio, Rebecca C. Wade

**Affiliations:** 1 Molecular and Cellular Modeling Group, Heidelberg Institute for Theoretical Studies (HITS), Schloss-Wolfsbrunnenweg 35, 69118 Heidelberg, Germany; 2 Institute of Structural Biology, Helmholtz Zentrum München, Ingolstädter Landstr. 1, 85764 Neuherberg, Germany; 3 Munich Center for Integrated Protein Science, Biomolecular NMR Spectroscopy, Department Chemie, Technische Universität München, Lichtenbergstrasse 4, 85747 Garching, Germany; 4 Laboratoire d’Optique et Biosciences, Ecole Polytechnique, CNRS UMR7645, INSERM U1182, Université Paris-Saclay, 91128, Palaiseau, France; 5 Université Paris-Sud, 91405, Orsay, France; 6 Center for Molecular Biology (ZMBH), DKFZ-ZMBH Alliance, Heidelberg University, 69120 Heidelberg, Germany; 7 Interdisciplinary Center for Scientific Computing (IWR), Heidelberg University, Heidelberg, Baden-Württemberg, Germany

## Abstract

Conservation of function across families of orthologous enzymes is generally accompanied by conservation of their active site electrostatic potentials. To study the electrostatic conservation in the highly conserved essential enzyme, thymidylate synthase (TS), we conducted a systematic species-based comparison of the electrostatic potential in the vicinity of its active site. Whereas the electrostatics of the active site of TS are generally well conserved, the TSs from minimal organisms do not conform to the overall trend. Since the genomes of minimal organisms have a high thymidine content compared to other organisms, the observation of non-conserved electrostatics was surprising. Analysis of the symbiotic relationship between minimal organisms and their hosts, and the genetic completeness of the thymidine synthesis pathway suggested that TS from the minimal organism *Wigglesworthia glossinidia* (W.g.b.) must be active. Four residues in the vicinity of the active site of *Escherichia coli* TS were mutated individually and simultaneously to mimic the electrostatics of W.g.b TS. The measured activities of the *E. coli* TS mutants imply that conservation of electrostatics in the region of the active site is important for the activity of TS, and suggest that the W.g.b. TS has the minimal activity necessary to support replication of its reduced genome.

The electrostatic potential of a protein plays a crucial role in steering ligands to their binding sites, and orienting them correctly for binding^1^. In enzymes, the active site electrostatic potential is important for stabilizing the transition state and thereby catalyzing the reaction^2^. Therefore, conservation of protein function across a protein family is often accompanied by conservation of the electrostatic potential in the active site region, even though the rest of the protein may lack a conserved electrostatic potential^3^,^4^. Consequently, comparison of protein electrostatic potentials has been employed as a tool to predict protein function and to derive similarities in protein function across protein families^5^,^6^,^7^. Optimizing the electrostatic complementarity between a ligand and the binding site of a protein is also an important aspect in drug design^8^,^9^ and may provide a route to gain target selectivity^10^.

Owing to the importance of electrostatics in the function of enzymes, and our interest in the highly conserved essential enzyme, Thymidylate synthase (TS)^11^, we analyzed the conservation of electrostatics at the binding site of TS in various organisms. TS catalyzes the sole pathway for *de novo* synthesis of deoxythymidine monophosphate (dTMP) from deoxyuridine monophosphate (dUMP) and 5,10-methylenetetrahydrofolate (mTHF)^11^. dTMP serves as a precursor for synthesis of deoxythymidine triphosphate (dTTP) which is then incorporated into DNA. The only other route to obtain dTMP is by phosphorylating thymidine with thymidine kinase, in which case thymidine must be taken up by the cell from external sources. This route is limited by the availability of extracellular thymidine and the presence of nucleotide transporters.

The substrates and products of TS, dUMP, mTHF, dTMP and dihydrofolate, are charged molecules. Moreover, electrostatics have been shown to be important for channeling dihydrofolate from TS to dihydrofolate reductase (DHFR) in the bifunctional TS-DHFR proteins^12^. It was therefore of interest to compare the electrostatics of the binding site and examine any outliers for this functionally conserved enzyme. Here, we report a comparison of the electrostatic potential of the active site region of TS enzymes from 110 organisms using the PIPSA (Protein Interaction Property Similarity Analysis)^13^,^14^,^15^ procedure, which reveals the minimal organisms as an outlier class in contrast to the overall well-conserved electrostatics of the enzyme. Since a change in the electrostatic potential of the active site can alter the functional profile of an enzyme, and the genomes of minimal organisms are particularly rich in thymidine content (~70–80% AT content) compared to the other organisms^16^, the observation of a lack of electrostatic conservation in TS for this class of organisms led us to conduct an in-depth analysis of the significance of electrostatics for the activity of TS and the potential role of TS in the minimal organism *Wigglesworthia glossinidia brevipalpis* (W.g.b.).

## Results and Discussion

### Atypical electrostatic properties of thymidylate synthase enzymes from minimal organisms

**Figure 1:**
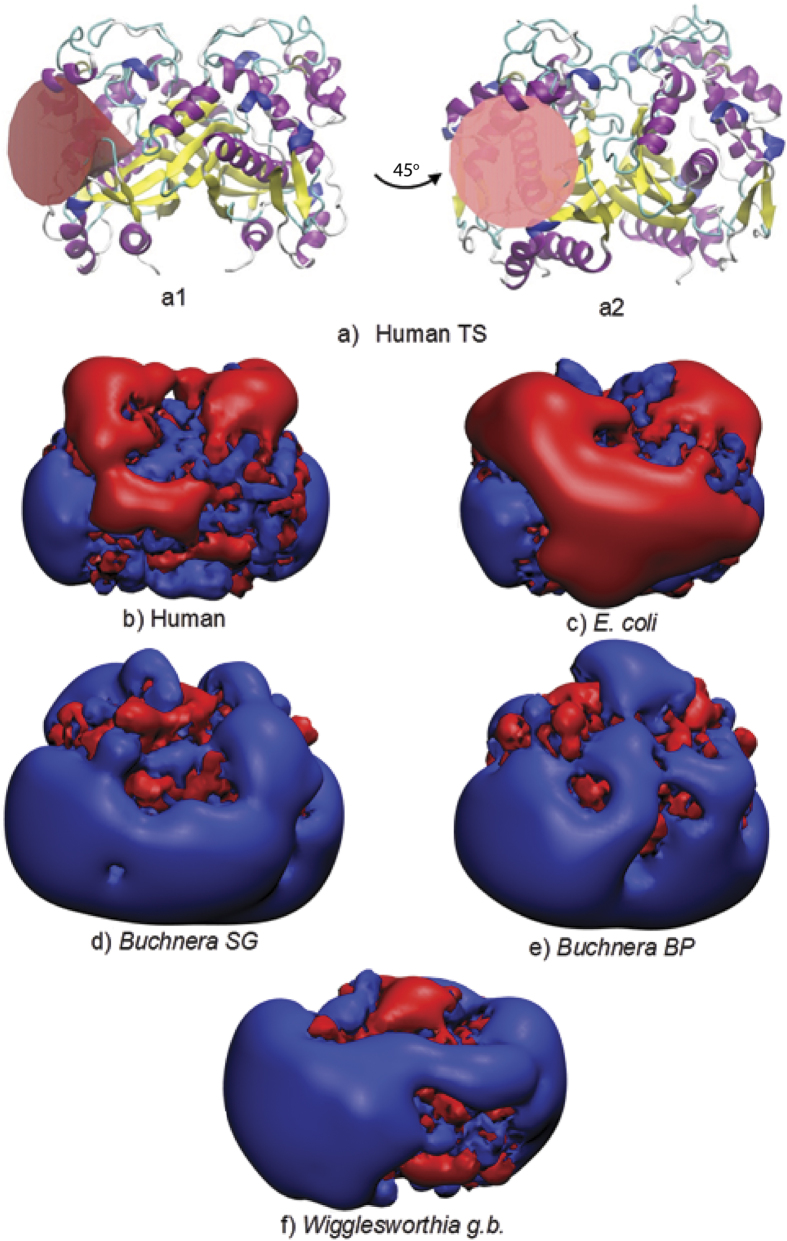
Electrostatic potentials of TS enzymes and definition of region for comparative analysis. (**a**) The conical region (red) defining the region for PIPSA comparison of the electrostatic potentials in the TS binding pocket, depicted in two orientations, a1 and a2, rotated by about 45°. The conical region has an aperture of 30° and its apex and axis direction are defined by the coordinates (Å) (47.713, −7.286, 38.419) and (48.759, −17.954, 35.932), respectively, for the coordinate file in Supplementary Information for human TS (P04818.pdb, for the UniProt sequence P04818 modelled on the crystal structure, 1HVY). (**b–f**) The electrostatic potentials of the TS enzymes from 5 organisms computed at 50 mM ionic strength and pH 7 are shown by the isocontours (blue +1 kT/e, red −1 kT/e). The orientation is the same as in a2.

**Figure 2:**
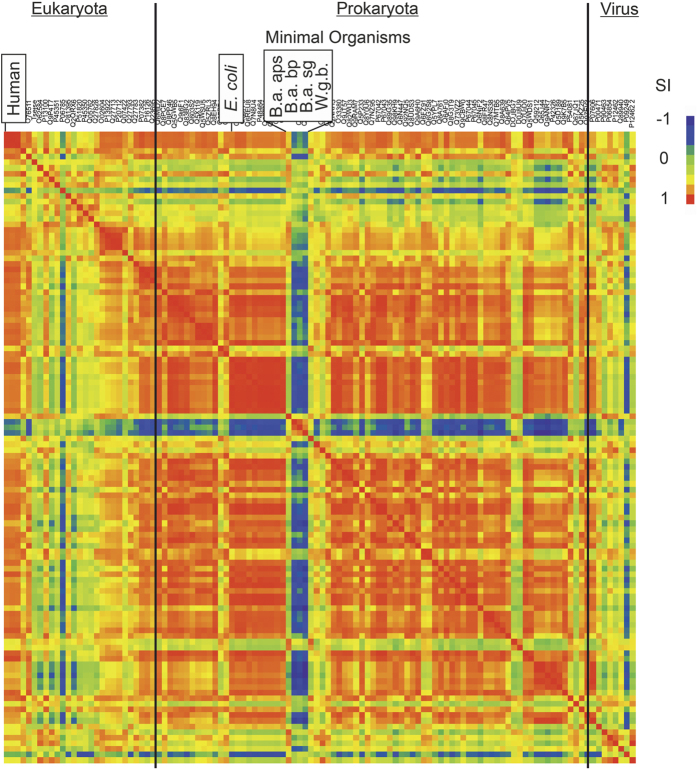
Heat map of pairwise similarity index (SI) values in the region of the active site (see Fig. 1a for definition) of the TS sequences modeled against the human TS (PDB ID: 1HVY). The sequences are arranged by the taxonomy of the organism and are labeled with their UniProt accession code. The human, *E. coli* and minimal organisms are boxed. Red indicates high similarity (SI = 1), green indicates uncorrelated potentials, and blue indicates opposite potentials (SI = −1).

To compare the electrostatic properties, the homodimeric structures of TS enzymes from 110 different organisms (listed in Supplementary Information) were modeled and their electrostatic potentials were computed (see Methods). Pairwise similarity indices (SI) for the protein electrostatic potentials in the region of the active site of one of the monomers of the modeled homodimeric TS structures (see Methods and Fig. 1 for definition of the region) were calculated using the PIPSA procedure^13^,^14^,^15^, and plotted as a heat map ordered by the annotated taxonomy of the organism from which the TS sequence was taken (Fig. 2). Upon visualization of the heat map, the dissimilar potentials of the minimal organisms, *Buchnera aphidicola subsp. Baizongia pistaciae*, *Buchnera aphidicola subsp. Schizaphis graminum* and W.g.b., were distinctly visible as vertical and horizontal blue-green stripes in the middle of the heat map, in contrast to the mostly red-yellow plot of pair-wise similarity indices, and in contrast to other prokaryotic organisms. The values of the similarity indices for these minimal organisms to other TSs were close to zero or negative, indicating unrelated or opposite electrostatic potentials, respectively. However, unexpectedly, they were positively correlated with each other (red square in the center of Fig. 2, and Table S1). For the purpose of comparison, human and *E. coli* TS enzymes were chosen as representatives for eukaryota and prokaryota, respectively. Visualization of the electrostatic isopotential surfaces revealed that the TS enzymes from minimal organisms are not only overall more positively charged but that they also have more positive electrostatic potentials in the active site regions compared to other TSs (Fig. 1). To investigate this surprising observation, a detailed analysis of TS in the minimal organisms was performed.

### Minimal organisms: A special case

The host-symbiont relationship in most cases of insect-obligate bacterial endosymbiont pairs is so strong that the bacteria cannot be cultured outside the host cells, rendering them difficult to study. Cellular domestication over millions of years has resulted in elimination of 70–75% of the ancestral genome in these bacteria^17^. Owing to the presence of nearly minimal gene sets, 450–800 kbp in length, these bacteria are called minimal organisms^18^,^19^,^20^. Unlike most free-living larger prokaryotes, which tend to be guanidine and cytosine (GC)-rich, a peculiar feature of the genetic content of minimal organisms is their high adenine and thymidine (AT) content (about 70–80%)^16^. In cells, the precursor for adenine incorporation into DNA, 2′-deoxyadenosine-5′-triphosphate (dATP) can be derived via multiple cross-linked pathways from hypoxanthine, inosine, adenine or their conjugates. The precursor for incorporation of thymidine, 2′-deoxythymidine-5′-triphosphate (dTTP), on the other hand, must be derived from dTMP, which may be derived by phosphorylating thymidine with thymidine kinase or by *de novo* synthesis from 2′-deoxyuridine-5′-monophosphate (dUMP) by TS (Fig. S1). Thus, the unusual electrostatic profile of the active site of TS is particularly interesting, since the activity of this enzyme could be crucial for supplying the levels of dTMP needed by the bacteria for their high AT content.

### Evidence for functionality of TS in the minimal organism W.g.b.

Minimal organisms tend to accumulate deleterious mutations due to the absence of robust DNA repair and recombination mechanisms. Moreover, they can depend on the host cell for a number of metabolic requirements^20^. Thus, the very different electrostatic potentials of the binding pocket of the TS enzymes from the minimal organisms could prompt one to think that these TS enzymes may have accumulated unfavorable mutations, and that the TS pathway might be on the route to deletion in these organisms. However, the following factors suggest that is not the case.

First, the mutations in the genome of minimal organisms tend to favor thymidine- and adenine-rich codons^20^. Thus, there is a cellular demand for dTMP precursors for DNA synthesis. If TS, the key enzyme for *de novo* dTMP synthesis, is dysfunctional or less active, the organisms may utilize the so-called salvage pathway (Fig. S1). However, the minimal organisms investigated in this study do not code for the key enzyme for the salvage pathway, thymidine kinase, making the use of this pathway unlikely. Therefore, unless an alternate but so far unknown pathway or transporter for procuring thymidylate exists, the TS in the minimal organisms must be functional.

The second aspect to be considered is the completeness of the thymidine synthesis pathway. Based on sequencing and annotation studies of the *Buchnera* and W.g.b. genomes, charts of the putative metabolic pathways in these organisms have been drawn^20^. Analysis of the thymidine biosynthesis pathway (Fig. S1) of *Buchnera* suggests that these organisms are able to synthesize UMP, but many enzymes required for its conversion to thymidine and dTTP are missing. The absence of some enzymes, particularly kinases, may be explained by relaxed substrate specificity of the enzymes present^21^. However, in the absence of an annotated means of dTTP synthesis or procurement, the functionality of TS in *Buchnera* will remain unclear. W.g.b., on the other hand, codes for the complete pathway for thymidine and dTTP biosynthesis.

Third, whereas the host aphids depend on *Buchnera* for certain amino acids^22^, W.g.b. supplies the tsetse fly with vitamins, including folate^23^. Since TS provides one of the routes of conversion of 5,10-methylene tetrahydrofolate to dihydrofolate, which can further be converted to folate, TS can contribute towards the sustenance of the symbiotic relationship of W.g.b. with its host, which is the vector of the parasite *Trypanosoma brucei* which causes the fatal disease, human African trypanosomiasis, also known as sleeping sickness.

Although it is not clear from these data whether the TS in minimal organisms is, in general, functional, these three lines of evidence indicate that TS is essential in W.g.b. and therefore should be functional.

### Four lysines are responsible for the deviant electrostatic potential in the ligand binding site of W.g.b. TS

**Figure 3:**
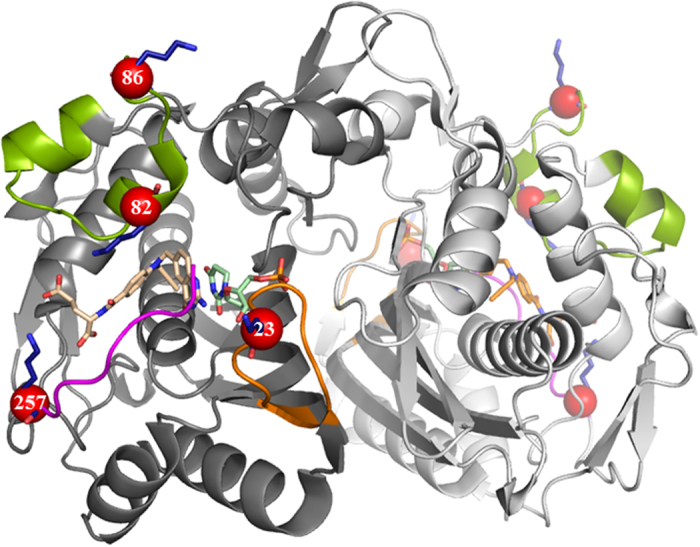
The locations of the mutated residues (red spheres) in the *E.coli* TS homodimeric structure (cartoon representation) (PDB ID 2G8O). The small domain is colored green, the R21 loop orange, and the C-terminus purple. The co-crystallized ligands are shown in stick representation: dUMP (pale green) and a folate analogue (beige). The modeled lysine residues at the mutated positions are shown in blue stick representation.

To identify the residues responsible for the high positive potential of W.g.b. TS, the active site of the homology model of W.g.b. TS, prepared using the crystal structure of human TS (PDB ID: 1HVY)^24^ as a template, was compared with the structures of human and *E. coli* TS enzymes in an active conformation. It was found that W.g.b. TS has four lysine residues at positions 23, 82, 86, and 257 which, if mutated to the corresponding residues of *E. coli*, i.e. G23, E82, E86, G257, make the potential of the binding pocket of the resulting W.g.b. TS quadruple mutant (W.g.b_mut) similar to that of the human and *E. coli* TS enzymes (Table S1). The length of the sequence of W.g.b. TS is the same as *E. coli* TS (264 residues), whereas human TS is longer (313 residues) with a large insertion at the N terminus, in the small domain (*E. coli* TS residues 69–88) and in the interface loop (*E. coli* TS residues 100–110) (Fig. S2). Therefore, additional homology models of the W.g.b. and W.g.b_mut TSs were made using the *E. coli* TS (PDB ID 2G8O)^25^ as a template, and subjected to PIPSA comparison. The results (Table 1) were similar to those obtained for models prepared using the human TS template (Table S1). Additionally, an *E. coli* TS quadruple mutant with residues G23, E82, E86, and G257 mutated to K, as in W.g.b., was modeled (*E. coli*_4K) (Fig. 3). The PIPSA comparison showed that its binding pocket was more similar to the W.g.b. TS than the *E. coli* TS (Table 1). Therefore, it was confirmed that the presence of lysine at the four positions, 23, 82, 86 and 257, was largely responsible for the dissimilarity of the electrostatic potential of the W.g.b. TS binding site compared to the other TSs analyzed. Because all the four identified positions were substituted with K in W.g.b. and not with a combination of the two positively charged amino acids, K and R, we carried out an analysis of the abundance of K in the W.g.b. proteome.

### General abundance of lysines in W.g.b.

Since minimal organisms tend to accumulate mutations favoring AT-rich codons, their proteins tend to be abundant in isoleucines (coded by ATT, ATA, ATC) and lysines (coded by AAA and AAG). The codon usage statistics obtained from the Microbial Genome Codon Usage Database (MGCUD) (http://bioinformatics.forsyth.org/mgcud) show that lysine codons account for 12% of the total number of W.g.b. codons as opposed to only 4% in *E. coli*. A similar trend is present in the amino acid content of TS, with lysine constituting 10% of the W.g.b. protein, but only 5% in *E. coli* TS. These observations imply that the higher abundance of lysines, and consequently, the positive potential, are not a specific feature of TS in W.g.b. but merely correspond to the general trend for all proteins in the organism. This also accounts for the presence of K instead of a mixture of both K and R at the selected positions. It is therefore reasonable to expect that, in spite of the presence of the lysines, which alter its electrostatic potential in the active site region, the TS in W.g.b. would be functional.

Considering that the four selected lysines are close to the active site (Fig. 3), the side chain of lysine is longer than that of glutamic acid or glycine (the corresponding residues in *E. coli*) and the residue flexibility is also different, it is possible that, in addition to electrostatic dissimilarity, these lysines will result in steric differences that may affect the activity of the TS in W.g.b. Thus, on the basis of the computational analysis and mutant design, we next carried out experimental assays to analyze the effect of lysine residues at the selected positions.

The recombinant W.g.b. TS expressed as inclusion bodies in *E. coli* based expression systems, and was therefore unsuitable for analysis. Instead, we used *E. coli* TS as a model system by mutating *E. coli* TS to mimic the electrostatic features of W.g.b. TS and then performing kinetic studies.

### Experimental characterization of lysine mutants of *E. coli* TS shows specific effects of lysines on enzyme activity

*E. coli* TS was mutated to simultaneously introduce lysines at the four positions, i.e. G23K/E82K/E86K/G257K (*E. coli*_4K) and this *E. coli*_4K mutant was used as a model to mimic the electrostatic features of W.g.b. TS (Table 1). The quadruple mutant failed to complement a ThyA negative *E. coli* strain (Table 2, Supplementary Fig. S3) but nevertheless showed some activity *in vitro* with similar k_cat_/K_m_ values to the wild-type (WT) protein for both substrates, but lower k_cat_ and K_m_ values.(Table 3).

To understand the contributions of the various mutated residues, each of the four positions in *E. coli* TS was individually mutated to lysine, resulting in four single point mutants. The results of the kinetic assays are given in Supplementary Fig. S4 and summarized in Table 3. While the E82K and G257K mutations resulted in reduced catalytic efficiency (k_cat_/K_m_) for both ligands, the k_cat_/K_m_ values of the G23K mutant increased when compared to the WT protein under identical conditions. For the E86K mutant, on the other hand, the k_cat_/K_m_ values were similar to that of the WT enzyme. Since the G23K and E86K mutants do not have as detrimental effects on the catalytic efficiency of the enzyme as E82K and G257K, a double mutant, G23K/E86K (*E. coli*_2K), was made to further characterize their effect. The k_cat_/K_m_ value of the *E. coli*_2K double mutant was about the same as the WT for both ligands. A detailed structure, sequence and literature analysis was carried out to interpret these results, and is discussed in the following sections.

*G23K mutant.* The G23K mutation had the most surprising effect on activity. By reducing the K_m_ value for both substrates – dUMP and mTHF - the k_cat_/K_m_ value of this mutant was 1.4–2.6 times higher than that of the WT enzyme. The residue G23 is in the conserved motif DRT*G*TGT (Table 4 Align.1) in the loop formed by residues 18–28. This loop contains the residue R21, which makes a hydrogen bond with the phosphate of dUMP, thereby contributing to its binding stability^25^. Mutations in this loop are reported to make human TS resistant to 5-fluorodeoxyuridine^26^, suggesting altered binding of TS to the ligand. In *E. coli* TS, mutating G23 to uncharged residues such as S/N/Y/L does not alter the specific activity of the protein, however mutations to the charged residues H/E lead to about 70% reduction in specific activity^27^. Therefore, the observed decrease in the K_m_ value of dUMP and consequent increase in k_cat_/K_m_ value for G23K was not anticipated. Nevertheless, K has the longest side chain and strongest positive charge compared to any of the other previously reported mutations at this site. Hence, the observed effect might be due to altered electrostatics and/or flexibility of the R21 loop which is involved in coordinating the dUMP phosphate group, together with a possible effect, due to its proximity (Fig. 3), on the opening and closing movement of the C-terminus. The TS protein is known to exist in two structural states - the open and the closed forms - distinguished by the movement of the C-terminus, and essential for the functioning of the enzyme^28^. Therefore, altered movement of this region can alter the activity of the enzyme.

*E82K mutant.* The E82K mutation was observed to have the most drastic effect with the k_cat_/K_m_ values at about 10% of those of the WT enzyme. Interestingly, while the k_cat_ values were reduced dramatically, the K_m_ values were almost unaltered for both dUMP and mTHF. Therefore, it is unlikely that the increased length of the side-chain of K as compared to E caused direct steric hindrance to the entry of the ligands into the binding pocket. In addition to strong electrostatic drift, the interaction of the positively charged side-chain of K82 with the neighboring negative or aromatic residues (corresponding sequence WD(E/K)^82^WADE^86^) via salt bridge formation or cation-pi bonding could alter the structure and flexibility of the small domain (Figs. 3 and S2). This could in turn reduce the k_cat_/K_m_ values of the enzyme. This hypothesis can be corroborated by the fact that in our sequence alignment, W.g.b. TS is the only sequence with a positively charged residue at position 82 while most other organisms have either a negatively charged residue (glutamic acid 57%, aspartic acid 2%) or an uncharged residue (asparagine 33%, glutamine 5%) (Table 4 Align. 2).

*E86K mutant.* The E86K mutation displayed increased k_cat_ and K_m_ values for both ligands, resulting in an overall similar k_cat_/K_m_ values to the WT. Compared to E82, E86 is distant from the binding pocket (Fig. 3). Therefore, a direct steric hindrance resulting in increased K_m_ values is improbable. However, like E82, E86 is also present in the small domain, and a putative salt bridge interaction between the mutant K86 and D85 could alter the structure and flexibility of the small domain with consequent effects on ligand binding and the k_cat_/K_m_ values of the protein. Although position 86 is mostly conserved as a negatively charged residue (glutamic acid 65%, aspartic acid 10%) in the analyzed TS sequences, 3% of the TS sequences have a lysine at this position, indicating a slightly higher tolerance for lysine at position 86 than at position 82.

*G257K mutant.* Other than E82K, G257K was the only mutant which demonstrated reduced catalytic efficiency values for both ligands. The k_cat_ value for dUMP was decreased and K_m_ value for mTHF was increased. For both substrates, the result was reduced k_cat_/K_m_ ratios. Although the position 257 is not conserved in TS sequences, the G257K mutation might reduce the flexibility of the C-terminus (Fig. 3). Moreover, the side-chain of K257 can potentially form a salt bridge with the carboxylate of the folate cofactor, also resulting in reduced flexibility of the C-terminus and possibly some deviation in the binding mode of the ligand. Either or all of these factors could be responsible for the reduced k_cat_ value for dUMP and increased K_m_ value for folate, resulting in an overall decrease in enzyme activity.

*E. coli_2K double mutant.* For both substrates, the k_cat_/K_m_ values of the double mutant G23K/E86K TS protein were similar to the WT. These results were in agreement with the PIPSA results where *E. coli*_2K was observed to be electrostatically similar to *E. coli* WT (SI = 0.8), but more distant from *E. coli*_4K (SI = 0.2) and W.g.b. (SI = −0.2) (Table 1). Further, in cell complementation assays, the *E. coli*_2K mutant could partially compensate for the absence of WT TS (Table 2, Supplementary Fig. S3).

*E. coli_4K quadruple mutant.* In the case of the quadruple G23K/E82K/E87K/G257K TS mutant, the k_cat_ and K_m_ values for both substrates were reduced, resulting in overall similar k_cat_/K_m_ values as compared to WT TS. Taking the experimental and computational results for all the mutants together, it can be concluded that the E82K and G257K mutations are primarily responsible for the reduction in k_cat_ values of the quadruple *E. coli*_4K TS mutant. Although precise values could not be obtained, the K_m_ values of the *E. coli*_4K TS mutant were clearly lower than for any of the other mutants. These K_m_ values may reflect greater binding affinity of the mutant to the negatively charged substrates due to greater charge complementarity. The net charge of the mutant is +6e higher than the WT on each monomer of the TS homodimer. The possible allosteric effects discussed for the individual mutants may also act synergistically in the *E. coli*_4K TS mutant. The non-additive effect of the four simultaneous mutations may affect the kinetics of binding, possibly increasing the association rate constants for substrates. The four mutations could also influence substrate channeling between TS and dihydrofolate reductase, which may occur even though these are not expressed on the same polypeptide chain in either *E. coli* or W.g.b.^29^ Considering these kinetic data, the symbiotic lifestyle of W.g.b., the presence of a complete thymidine biosynthesis pathway, and the overall skewed amino acid composition coded by the W.g.b. transcriptome, we expect that the TS in W.g.b. should be active. The inability of the *E. coli*_4K TS mutant to complement the *thyA* negative *E. coli* strain and the lower measured k_cat_ values indicate, however, that W.g.b. TS may have a lower activity level than WT *E. coli* TS.

## Conclusions

The electrostatic potential at the binding site of the enzyme thymidylate synthase is largely conserved across various species from eukaryota, prokaryota and viruses. However, the minimal organisms in prokaryota do not conform to this trend, and demonstrate a large positive potential in the active site region. This distinct positive potential can be attributed to the high AT content of the genome of the minimal organisms and consequent high abundance of lysines in their proteome. A detailed study of the minimal organisms revealed that, among the minimal organisms in our dataset, the TS from W.g.b. is most likely to be functional. Therefore, the TS from W.g.b. provided an opportunity to investigate the significance of electrostatics for the activity of TS. By employing TS from *E. coli* as a model system and mutating four residues in the vicinity of the active site to mimic the electrostatic properties of TS from W.g.b., we find that the active site electrostatics are indeed crucial for the optimal activity of the TS enzyme. The results of *in vitro* kinetic and cellular complementation assays imply that W.g.b. TS should possess the minimal activity necessary to support the reduced genome of this endosymbiotic bacterium.

## Materials and Methods

### Materials

All chemical reagents were purchased from Sigma Aldrich except for mTHF, which was generously provided by R. Moser (Merck Eprova AG).

## Methods

### Sequence analysis and comparative modeling

The sequences of TS and dihydrofolate reductase (DHFR)-TS were retrieved from UniProt. For the cases where the DHFR and TS occur in the same polypeptide chain, the sequence corresponding to the DHFR and the linker sequence was removed, leaving only the TS sequence. A multiple sequence alignment was performed, using default parameters in ClustalW (1.83)^30^, against the protein sequence retrieved from the crystal structure of human TS (hTS), PDB ID 1HVY^24^. Sequences with less than 35% pairwise identity to hTS, or insertions of more than 8 residues, were removed. Homology models for 110 homodimeric proteins were built using Modeller9v1^31^ with the automodel class. The homodimer defined by chains A and B in protein structure 1HVY was used as the template structure. The models generated were subjected to a rapid optimization with conjugate gradients, as implemented in the ‘very_fast’ method available in automodel. The ligands were not added to the modeled structures. Homology models of the W.g.b., W.g.b_mut (K23G/K82E/K86E/ K257G), *E. coli*_2K(G23K/E86K) and *E. coli*_4K (G23K/E82K/E86K/G257K) TSs were also made using the *E. coli* TS (PDB ID 2G8O)^25^ as a template.

### Electrostatic calculations

The electrostatic potentials were calculated using the APBS package^32^. Prior to the electrostatic potential calculation, the atomic coordinates of the models of the TS homodimers were superimposed by sequence alignment-based matching of the Cα atoms using the in-house sup2pdb program, hydrogen atoms were added using the pdb2pqr software^33^, assuming standard protonation states at pH 7, and the models were optimized with the debump option to remove any unfavorable steric contacts to hydrogen atoms. The partial atomic charges and the radii were assigned using the AMBER99 force-field^34^. The relative dielectric constants of the solute and the solvent were set to 1 and 78, respectively. The dielectric boundary was defined by the protein’s van der Waals surface. The ionic strength of the solvent was set to 50 mM and the temperature was set to 298.15 K. The electrostatic potentials of the TS homodimers were computed by solving the linearized Poisson-Boltzmann equation using a single Debye-Hückel sphere boundary condition on a 65 × 65 × 65 grid with a spacing of 1.5 Å centered at the same point for all the proteins.

### Comparison of protein electrostatic potentials

The computed potentials were compared using Protein Interaction Property Similarity Analysis (PIPSA)^13^,^15^. A conical region was used to define the active site region in the chain A of the homodimeric protein over which the potentials were compared (See Fig. 1 and supplied coordinate files). When superimposed, the two subunits in the homodimer of the structure of the human WT TS (PDB ID: 1HVY)^24^ display a very low non-hydrogen-atom RMSD of 0.2 Å. Therefore, the two active sites were considered to be very similar, and analysis of one active site was considered sufficient. Hodgkin similarity indices^35^,^36^ (SI), were computed for each pair of proteins (labeled 1 and 2) using equations 1 and 2:


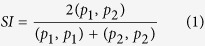



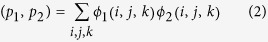


where (p1, p2), (p1, p1) and (p2, p2) are the scalar products of the protein electrostatic potentials over the region where the potentials are compared, ϕ is the protein electrostatic potential, and (i, j, k) are the grid points that are within the region of interest, the overlapping ‘skins’ of the two proteins within the conical region. Each protein skin has a of thickness of 4 Å and is defined by the region accessible to the center of a probe of radius of 3 Å rolled over the protein and inaccessible to a probe of radius 7 Å. Thus SI = 1 if, in the region compared, the two potentials are identical; SI = 0 if they are uncorrelated; SI = −1 if they are anti-correlated. SI values were computed for all protein pairs and these were then analysed using a heat map (Fig. 2).

### Cloning and site-directed mutagenesis

The *thyA* gene was amplified from *E. coli* strain MG1655 DNA with the following pair of primers (sequences in small characters correspond to the genome sequence): *thyAfw* (CGGGATCCatgaaacagtatttagaactg) and *thyArev* (CCCAAGCTTttagatagccaccggcgcttt). The resulting PCR product was then digested with *Bam*HI and *Hin*dIII and cloned into pQE80L (Qiagen) to generate pQE80L_EcoliWT. The *thyA* genes encoding the double and quadruple mutants were synthesized by GeneCust Europe and cloned into the *Bam*HI and *Hin*dIII sites of pQE80L to give pQE80L_Ecoli_2K (G23K/E86K) and pQE80L_Ecoli_4K (G23K/E82K/E86K/G257K), respectively. Plasmids for single mutants were prepared by mutagenesis done following the QuikChange Site-Directed mutagenesis kit protocol (Stratagene) using the plasmid pQE80L_Ecoli_WT as a template. The primer couples designed for each mutation are given in Table S2. After mutagenesis, plasmids were sequenced to confirm the induced mutation.

### Complementation tests

FE013 (Δ*thyA::aphA3*), an *E. coli* strain carrying a deletion of *thyA*, was transformed with pQE80L, pQE80L_Ecoli_WT, pQE80L_Ecoli_2K and pQE80L_Ecoli_4K plasmids^37^. Transformants were selected at 37 °C on Luria Broth agar plates with 100 μg/ml ampicillin and 20 μg/ml kanamycin. Each of the different strains was then streaked onto M9 minimal medium plates containing 0.2% casamino acids and supplemented with or without 0.5 mM IPTG (isopropyl-beta-D-thiogalactopyranoside). Growth was scored by observing the colonies after incubation for 3 days at 37 °C.

### Protein expression and purification

WT *E. coli* TS and all mutants were expressed in *E. coli* BL21(DE3) cells. Transformed bacteria were grown in standard Luria Broth culture medium at 37 °C and genetic selection was made by adding ampicillin (100 mg/mL). Protein expression was induced at OD_600_ = 0.6 – 0.8 with 0.4 mM IPTG (isopropyl-beta-D-thiogalactopyranoside). After 4 hours, cells were harvested by centrifugation at 5000 × g for 20 min at 4 °C, and the cell pellets were frozen at −20 °C. Cell lysis was done by repeated freezing and thawing, followed by sonication in lysis buffer (50 mM Tris pH 7.5, 300 mM KCl, 25 mM imidazole, 1 tablet of Complete Protease Inhibitor (Roche)) and followed by centrifugation for 20 min at 20,000 × g at 4 °C. Protein purification was carried out by using Protino® Ni-IDA packed columns based on IDA (iminodiacetic acid); proteins were eluted with 50 mM sodium phosphate buffer, 300 mM NaCl, 250 mM imidazole, pH 8.0. To eliminate imidazole, proteins were loaded in PD-10 (Sephadex G-25 Medium) desalting columns and eluted with 50 mM sodium phosphate buffer, pH 8.0, 300 mM NaCl. Protein concentration was determined from absorption measurements at 278 nm using the absorption coefficient of 1.17 × 10^5^ M^−1^cm^−1^. The purified protein was stored at –80 °C.

### Enzymatic activity assays

TS enzymatic activity was determined spectrophotometrically by monitoring the increase in absorbance at 340 nm during the oxidation reaction of mTHF to 7,8-dihydrofolate. Varying concentrations of the substrates were mixed with purified TS in 20 mM phosphate buffer, pH 6.9, at 25 °C and aliquots of this mixture were assayed for TS activity under standard conditions: an aliquot of enzyme (0.5–1 μg/mL depending on the mutant specific activity), was added to 1 mL of assay buffer consisting of 50 mM TES, pH 7.4, containing 25 mM MgCl_2_, 6.5 mM HCHO, 1 mM EDTA, 75 mM β-mercaptoethanol, and variable dUMP and mTHF concentrations. Following the addition of the substrate, the absorbance was monitored at 340 nm in a UV-visible spectrophotometer for 3 minutes. The k_cat_ and the Michaelis-Menten constant (K_m_) of the mutants was determined for mTHF and dUMP by varying the concentration of either substrate from 0 to 300 μM in the presence of a fixed concentration of 200 μM of the other substrate.

## Additional Information

**How to cite this article**: Garg, D. *et al.* Conservation and Role of Electrostatics in Thymidylate Synthase. *Sci. Rep.* **5**, 17356; doi: 10.1038/srep17356 (2015).

## Supplementary Material

Supplementary Information

Dataset 1

Dataset 2

## Figures and Tables

**Table 1:** Hodgkin similarity indices calculated for *E. coli* TS, W.g.b. TS and their mutants modeled using the *E. coli* TS structure (PDB ID 2G8O) as a template.

	*E. coli*	*E. coli*_2K*	*E. coli*_4K†	W.g.b	W.g.b_mut‡
*E. coli*	1	0.819	0.052	−0.387	0.454
*E. coli*_2K*	0.819	1	0.192	−0.151	0.568
*E. coli*_4K†	0.052	0.192	1	0.731	0.532
W.g.b	−0.387	−0.151	0.731	1	0.325
W.g.b_mut‡	0.454	0.568	0.532	0.325	1

^*^E. coli_2K: double mutant, G23K/E86K.

^†^*E. coli*_4K: quadruple mutant, G23K/E82K/E86K/G257K.

^‡^W.g.b_mut: W.g.b. TS with four lysines mutated to the corresponding *E. coli* residues, K23G/K82E/K86E/K257G.

**Table 2:** Results of cell complementation tests for ThyA-negative *E. coli* FE013 (pQE80L) as a control, and for pQE80L expressing the WT TS (*E. coli*_WT), the *E. coli* double mutant G23K/E86K (*E. coli*_2K), and the *E. coli* quadruple mutant G23K/E82K/E86K/G257K TS (*E. coli*_4K), see Fig. S3.

Strain	Without IPTG	With IPTG
FE013 (pQE80L)	–	–
FE013 (pQE80L_*E. coli*_WT)	++§	+++
FE013 (pQE80L_*E. coli*_2K)	+/−§	+
FE013 (pQE80L_*E. coli*_4K)	–	–

^§^Complementation of the *E. coli* Δ*thyA* strain under the conditions of leaky expression in the absence of the IPTG inducer was observed for the wild type *thyA* gene and, to a very limited extent, for the double mutant *thyA* gene. Furthermore, complementation was observed for all single lysine mutants under similar conditions of leaky expression (data not shown).

**Table 3:** Results of kinetic assays for *E. coli* WT and mutant TS enzymes.

	dUMP			mTHF		
	k_cat_ (s^−1^)	K_m_ (μM)	k_cat_/K_m_(s^−1^μM^−1^)	k_cat_ (s^−1^)	K_m_ (μM)	k_cat_/K_m_(s^−1^μM^−1^)
WT^¶^	0.32 ± 0.02	82 ± 9	0.004 ± 0.0005	0.28 ± 0.01	77 ± 8	0.004 ± 0.0004
WT^║^	0.60 ± 0.33	58 ± 9	0.01 ± 0.002	0.48 ± 0.04	59 ± 14	0.008 ± 0.002
Ratio^**^	**mutant/WT**	**mutant/WT**	**mutant/WT**	**mutant/WT**	**mutant/WT**	**mutant/WT**
G23K^¶^	0.94 ± 0.07	0.37 ± 0.07	2.56 ± 0.54	0.86 ± 0.08	0.64 ± 0.15	1.35 ± 0.33
E82K^¶^	0.16 ± 0.03	1.10 ± 0.44	0.14 ± 0.06	0.11 ± 0.04	1.19 ± 0.81	0.09 ± 0.07
E86K^║^	2.17 ± 0.27	2.41 ± 0.80	0.90 ± 0.32	1.67 ± 0.40	2.54 ± 1.32	0.66 ± 0.37
G257K^║^	0.57 ± 0.06	0.86 ± 0.23	0.66 ± 0.19	1.10 ± 0.19	2.71 ± 1.09	0.41 ± 0.18
*E. coli*_2K^║^	1.83 ± 0.44	1.86 ± 1.09	0.98 ± 0.62	1.88 ± 0.56	2.37 ± 1.61	0.79 ± 0.59
*E. coli*_4K^¶^	0.25 ± 0.06	~0.1	~1	0.21 ± 0.04	~0.2	~1

^¶^and ^║^ refer to independent sets of experiments and comparison within that set. See Fig. S4 for measured kinetic activities. Only approximate K_m_ values could be obtained for the *E. coli*_4K mutant as they fell outside the measurable range.

^**^Standard error for the ratio
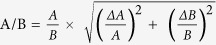

**Table 4:** Parts of the multiple sequence alignment of TS showing two conserved motifs, each containing an unusual lysine in W.g.b. K23 and K82 (bold font) are two of the four lysines responsible for the deviant electrostatic potential of W.g.b. For full alignment, see Fig. S2.

	Align. 1	Align. 2
Human	49 DRTGTGT 55	108 IWDANGS 115
*E. coli*	20 DRTGTGT 26	79 IWDEWAD 85
W.g.b.	20 DRT**K**TGT 26	79 IWN**K**WAD 85
