# Supplementary material for: Conservation and Role of Electrostatics in Thymidylate Synthase
Source: Sci Rep. 2015 Nov 27;5:17356. doi: 10.1038/srep17356 (PMC4661567; doi:10.1038/srep17356)
Supplement: Supplementary Information [file srep17356-s1.pdf]

Supplementary Information

Conservation and Role of Electrostatics in Thymidylate Synthase

Divita Garg[a,b,c,1], Stephane Skouloubris[d,e], Julien Briffotaux[d], Hannu Myllykallio[d], Rebecca C. Wade[a,f,g]

[a]Molecular and Cellular Modeling Group, Heidelberg Institute for Theoretical Studies (HITS), Schloss-Wolfsbrunnenweg 35, 69118 Heidelberg, Germany; [b]Institute of Structural Biology, Helmholtz Zentrum München, Ingolstädter Landstr. 1, 85764 Neuherberg, Germany; [c]Munich Center for Integrated Protein Science, Biomolecular NMR Spectroscopy, Department Chemie, Technische Universität München, Lichtenbergstrasse 4, 85747 Garching, Germany; [d]Laboratoire d'Optique et Biosciences, INSERM U1182, Centre National de la Recherche Scientifique Unité Mixte de Recherche 7645, Ecole Polytechnique, Palaiseau Cedex, France; [e]University of Paris-Sud, Orsay, F-91405; [f]Center for Molecular Biology (ZMBH), Heidelberg University, 69120 Heidelberg, Germany; [g] Interdisciplinary Center for Scientific Computing (IWR), Heidelberg University, Heidelberg, Baden-Württemberg, Germany

[1] Present address: The Scripps Research Institute, Department of Molecular and Experimental Medicine, 10550 North Torrey Pines Road, MEM-131, La Jolla, CA 92037, USA

The Supplementary Information consists of

- 1 PDF document containing Tables S1 and S2, Figures S1-S4, and a list of the proteins studied, and
- Two coordinate files in pqr format: P04818.pqr for human TS, and P0A886.pqr for *E. coli* TS. The coordinate files have hydrogen atoms added, atomic partial charges and radii for the electrostatic potential calculations, and give the coordinate system for defining the region for the PIPSA analysis defined in Figure S1. The human and *E. coli* TS structures were used as templates for homology modeling. The residue numbering scheme in these files corresponds to those of the corresponding PDB files, 1HVY (human) and 2G8O (*E.coli*).

Table S1. Values of the Hodgkin similarity index for the electrostatic potential at the active site of TS of various organisms modeled against the crystal structure of human TS (PDB ID 1HVY). Human and *E. coli* TS were chosen as representatives of eukaryotes and prokaryotes, respectively.

	P04818 (human)	P0A886 (*E. coli*)	Q8D2N4 (W.g.b)	P59427 (B.b.p)	Q8K9C3 (B.s.g)	W.g.b_ mut
P04818	1	0.849	-0.080	-0.088	-0.002	0.635
P0A886	0.849	1	-0.466	-0.320	-0.254	0.389
Q8D2N4	-0.080	-0.466	1	0.794	0.851	0.363
P59427	-0.088	-0.320	0.794	1	0.930	0.552
Q8K9C3	-0.002	-0.254	0.851	0.930	1	0.593
W.g.b_mut	0.635	0.389	0.363	0.552	0.593	1

Wigglesworthia glossinidia brevipalpis (W.g.b.), *Buchnera aphidicola subsp. Baizongia pistaciae* (B.b.p), and *Buchnera aphidicola subsp. Schizaphis graminum* (B.s.g). W.g.b_mut=W.g.b TS quadruple mutant K23G/K82E/K86E/K257G

Table S2. Primer sequences used to mutate the pQE80L_EcoliWT to code the four single mutants of *E. coli* TS.

**Mutation**		**Primer Sequence**
G23K	Forw.	cagaaaaacgaccgtaccAAAaccggaacgctttccat
	Rev.	atggaaagcgttccggtTTTggtacggtcgtttttctg
E82K	Forw.	aatgtcaccatctgggacAAAtgggccgatgaaaacg
	Rev.	cgttttcatcggcccaTTTgtcccagatggtgacatt
E86K	Forw.	tgggacgaatgggccgatAAAaacggcgacctcgggc
	Rev.	gcccgaggtcgccgttTTTatcggcccattcgtccca
G257K	Forw.	aaggctacgatccgcatccgAAAattaaagcgccggtggctat
	Rev.	atagccaccggcgctttaatTTTcggatgcggatcgtagcctt

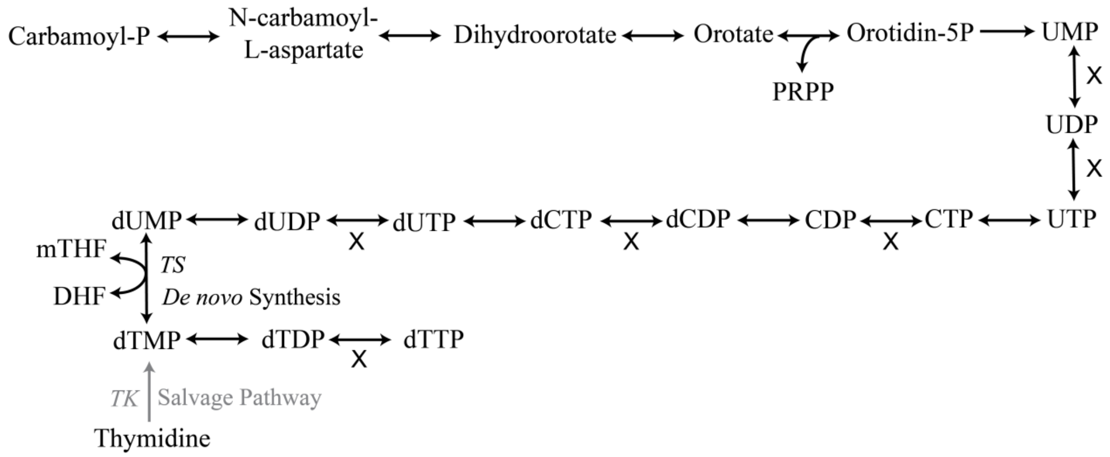

Figure S1. Representative thymidine synthesis pathway and thymidine salvage pathway. Black arrows represent the steps that are catalyzed by proteins whose coding sequences are present in the W.g.b. genome, whereas crosses denote the steps whose enzymes are lacking coding sequences in the *Buchnera* genomes. The grey arrow represents the step missing in both W.g.b. and *Buchnera* due to the lack of a coding sequence for thymidine kinase. The *de novo* synthesis of dTMP catalyzed by thymidylate synthase (TS) and the salvage pathway catalyzed by thymidine kinase (TK) are labeled.

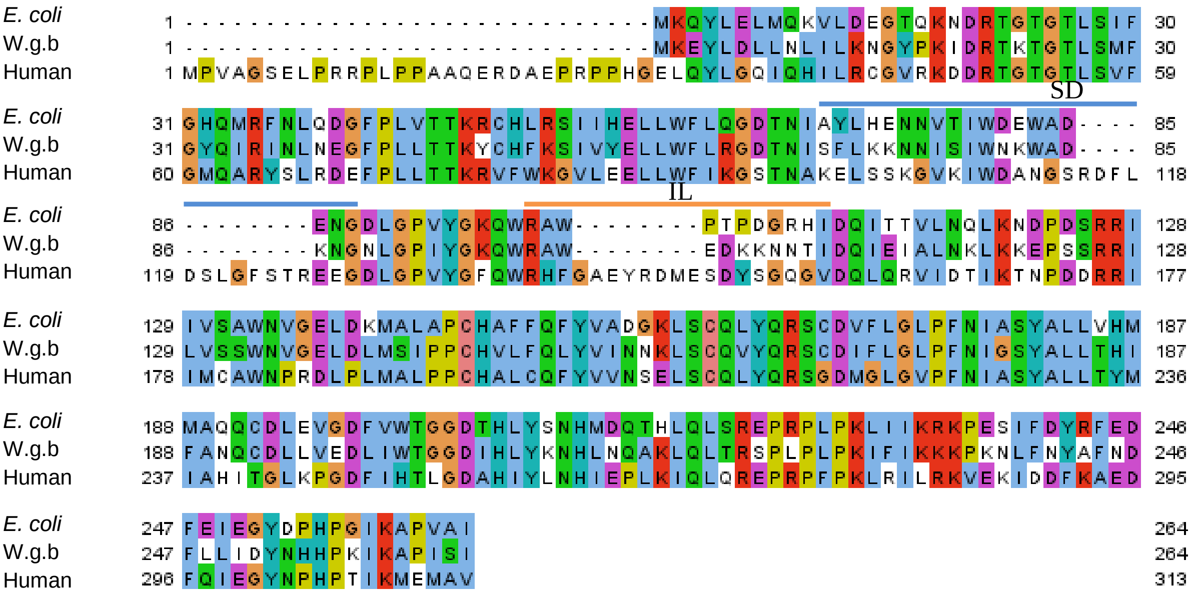

Fig S2. Sequence alignment of *E. coli,* W.g.b. and human TS proteins. The sequences are from UniProt (*E. coli*: P0A886; W.g.b.: Q8D2N4; human: P04818) and numbered accordingly. The background colors of the letters indicate residue type. The Small Domain (SD) and the Interface Loop (IL) are marked by horizontal blue and orange lines, respectively.

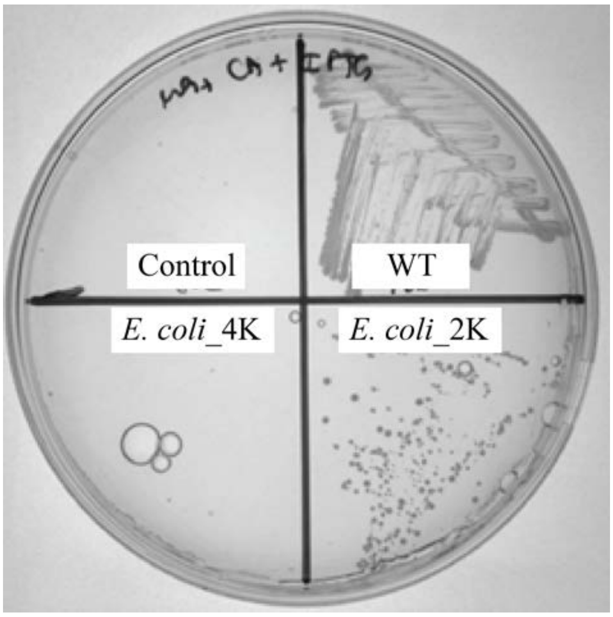

Fig S3. Cell complementation tests for ThyA-negative *E. coli* FE013 (pQE80L) as a control, and for pQE80L expressing the WT TS (*E. coli*_WT), the *E. coli* double mutant G23K/E86K (*E. coli*_2K), and the E. coli quadruple mutant G23K/E82K/E86K/G257K TS (*E. coli*_4K). See Table 2.

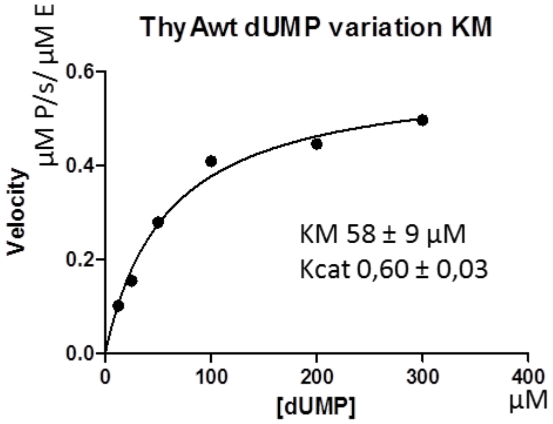
ThyAwt dUMP variation KM
Velocity
μM P/s/ μM E
KM 58 ± 9 μM
Kcat 0,60 ± 0,03
[dUMP]
μM

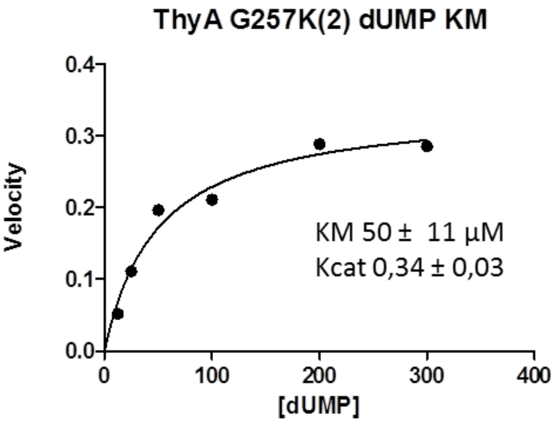
ThyA G257K(2) dUMP KM
Velocity
KM 50 ± 11 μM
Kcat 0,34 ± 0,03
[dUMP]

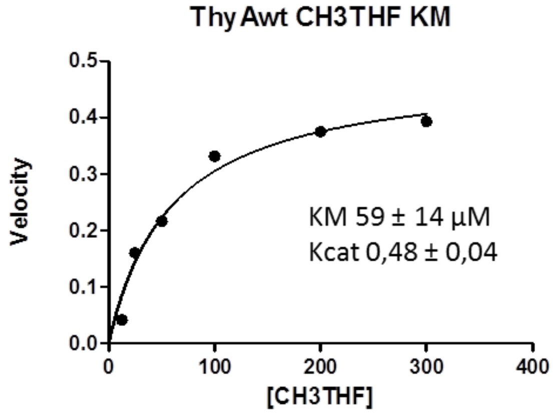
ThyAwt CH3THF KM
Velocity
KM 59 ± 14 μM
Kcat 0,48 ± 0,04
[CH3THF]

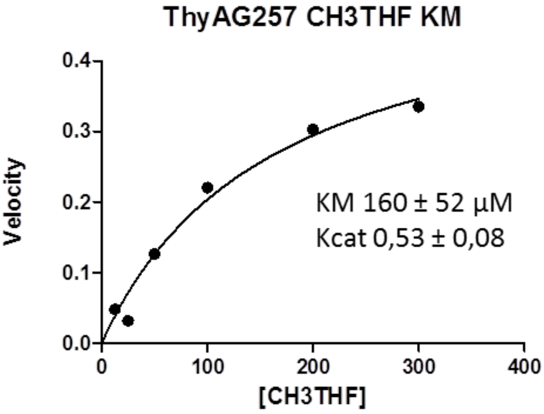
ThyAG257 CH3THF KM
Velocity
KM 160 ± 52 μM
Kcat 0,53 ± 0,08
[CH3THF]

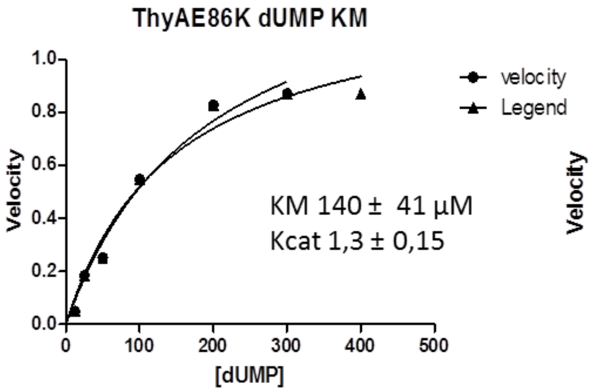
ThyAE86K dUMP KM
Velocity
velocity
Legend
KM 140 ± 41 μM
Kcat 1,3 ± 0,15
[dUMP]

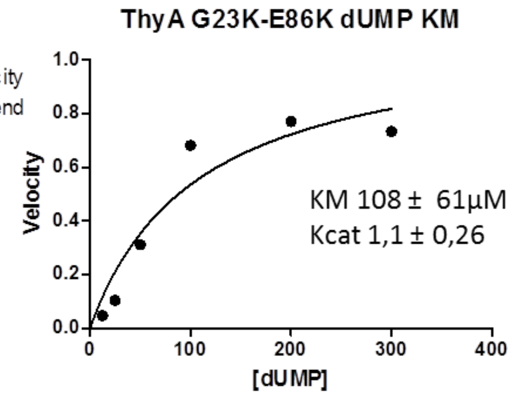
ThyA G23K-E86K dUMP KM
Velocity
KM 108 ± 61μM
Kcat 1,1 ± 0,26
[dUMP]

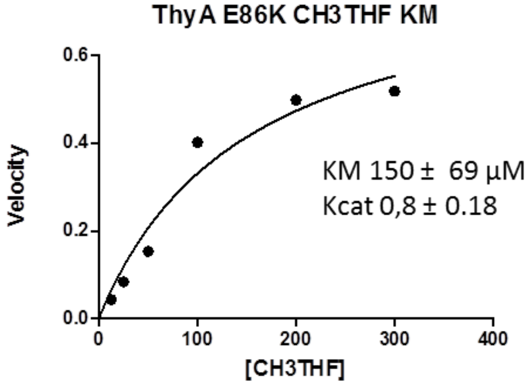
ThyA E86K CH3THF KM
Velocity
KM 150 ± 69 μM
Kcat 0,8 ± 0.18
[CH3THF]

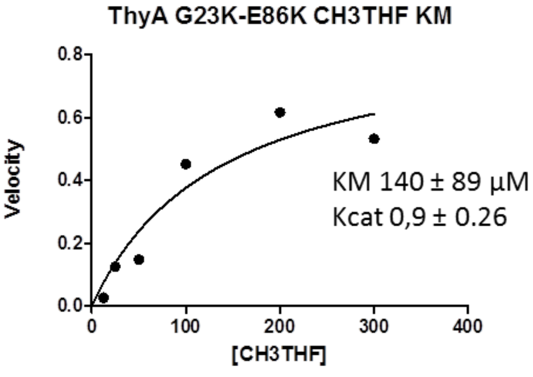
ThyA G23K-E86K CH3THF KM
Velocity
KM 140 ± 89 μM
Kcat 0,9 ± 0.26
[CH3THF]

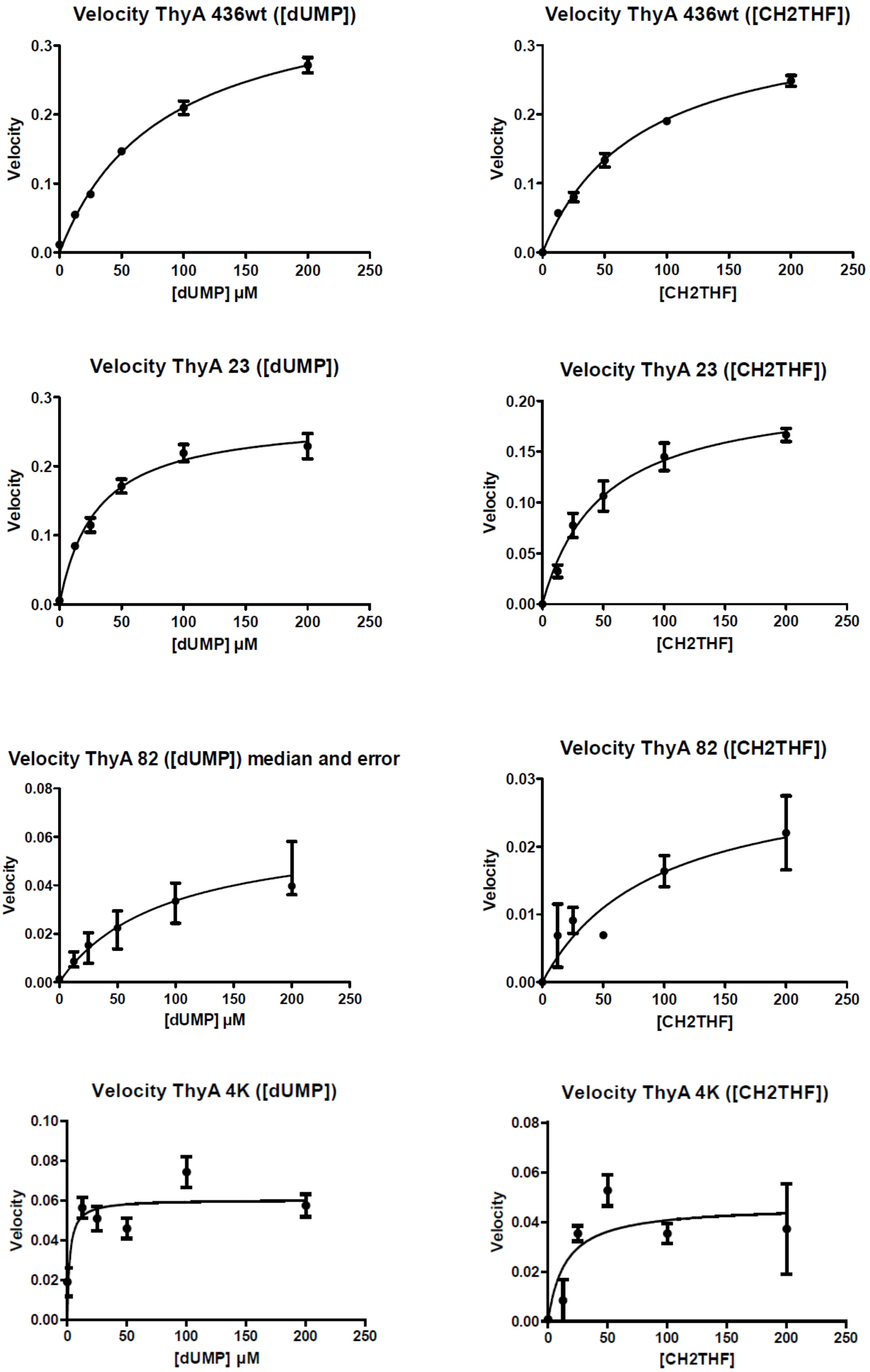

Fig S4. Measured kinetic activities for *E. coli* WT and mutant TS enzymes used to derive K_M and K_{cat} values given in Table 3. Error bars on measurements were derived from three independent measurements.

List of Uniprot IDs for the 110 TS proteins analysed, together with the organism name and taxonomy.
*The sequence for TS of tsetse fly was downloaded from the webpage of Prof. Serap Aksoy, Yale School of Public Health, U.S.A.

Eukaryota

P04818	*Homo sapiens*	Metazoa	Chordata
P45352	*Rattus norvegicus*	Metazoa	Chordata
P07607	*Mus musculus*	Metazoa	Chordata
O96650	*Ascaris suum*	Metazoa	Ecdysozoa
O76511	*Drosophila melanogaster*	Metazoa	Ecdysozoa
*	Tsetse fly	Metazoa	Ecdysozoa
O62584	*Encephalitozoon cuniculi*	Fungi	Microsporidia
P13100	*Pneumocystis carinii*	Fungi	Dikarya
Q9P4T7	*Agaricus bisporus*	Fungi	Dikarya
P45351	*Cryptococcus neoformans*	Fungi	Dikarya
P06785	*Saccharomyces cerevisiae*	Fungi	Dikarya
O81395	*Zea mays*	Viridiplantae	Streptophyta
Q2QRX6	*Oryza sativa*	Viridiplantae	Streptophyta
P51820	*Glycine max*	Viridiplantae	Streptophyta
P45350	*Daucus carota*	Viridiplantae	Streptophyta
Q05762	*Arabidopsis thaliana*	Viridiplantae	Streptophyta
Q27828	*Paramecium tetraurelia*	Alveolata	Ciliophora
O02604	*Plasmodium vivax*	Alveolata	Apicomplexa
P13922	*Plasmodium falciparum*	Alveolata	Apicomplexa
Q27713	*Plasmodium berghei*	Alveolata	Apicomplexa
P20712	*Plasmodium chabaudi*	Alveolata	Apicomplexa
Q07422	*Toxoplasma gondii*	Alveolata	Apicomplexa
Q27793	*Trypanosoma cruzi*	Euglenozoa	Kinetoplastida
Q27783	*Trypanosoma brucei*	Euglenozoa	Kinetoplastida
P07382	*Leishmania major*	Euglenozoa	Kinetoplastida
P16126	*Leishmania amazonensis*	Euglenozoa	Kinetoplastida
Q23695	*Crithidia fasciculata*	Euglenozoa	Kinetoplastida

Prokaryota

Q6MID2	*Bdellovibrio bacteriovorus*	Proteobacteria	Deltaproteobacteria
Q8PCE7	*Xanthomonas campestris*	Proteobacteria	Gammaproteobacteria
Q8PP46	*Xanthomonas axonopodi*	Proteobacteria	Gammaproteobacteria
Q5GWB5	*Xanthomonas oryzae*	Proteobacteria	Gammaproteobacteria
Q9I6F1	*Pseudomonas aeruginosa*	Proteobacteria	Gammaproteobacteria
Q83BG2	*Coxiella burnetii*	Proteobacteria	Gammaproteobacteria
Q603S2	*Methylococcus capsulatus*	Proteobacteria	Gammaproteobacteria
Q5X119	*Legionella pneumophila*	Proteobacteria	Gammaproteobacteria
Q5WSU5	*Legionella pneumophila*	Proteobacteria	Gammaproteobacteria
Q5ZRL3	*Legionella pneumophila*	Proteobacteria	Gammaproteobacteria
Q8EH94	*Shewanella oneidensis*	Proteobacteria	Gammaproteobacteria
Q87BT4	*Xylella fastidiosa*	Proteobacteria	Gammaproteobacteria

Q9PB13	*Xylella fastidiosa*	Proteobacteria Gammaproteobacteria
P0A886	*Escherichia coli*	Proteobacteria Gammaproteobacteria
Q8ZHV1	*Yersinia pestis*	Proteobacteria Gammaproteobacteria
Q667F9	*Yersinia pseudotuberculosi*	Proteobacteria Gammaproteobacteria
Q6REU8	*Xenorhabdus nematophilus*	Proteobacteria Gammaproteobacteria
Q7N8U4	*Photorhabdus luminescens*	Proteobacteria Gammaproteobacteria
P48464	*Shigella flexneri*	Proteobacteria Gammaproteobacteria
Q5PEN6	*Salmonella paratyphi-a*	Proteobacteria Gammaproteobacteria
Q8ZMA9	*Salmonella typhimurium*	Proteobacteria Gammaproteobacteria
Q8Z412	*Salmonella typhi*	Proteobacteria Gammaproteobacteria
Q6D8I6	*Erwinia carotovora*	Proteobacteria Gammaproteobacteria;
P57515	*Buchnera aphidicola*	Proteobacteria Gammaproteobacteria
P59427	*Buchnera aphidicola*	Proteobacteria Gammaproteobacteria
Q8K9C3	*Buchnera aphidicola*	Proteobacteria Gammaproteobacteria
Q8D2N4	*Wigglesworthia glossinidia brevipalpis*	Proteobacteria Gammaproteobacteria
P44420	*Haemophilus influenzae*	Proteobacteria Gammaproteobacteria
Q5R064	*Idiomarina loihiensis*	Proteobacteria Gammaproteobacteria
Q6FER7	*Acinetobacter sp*	Proteobacteria Gammaproteobacteria
Q2NRH3	*Sodalis glossinidius*	Proteobacteria Gammaproteobacteria
O33380,	*Neisseria gonorrhoeae*	Proteobacteria Betaproteobacteria
Q9JT57	*Neisseria meningitidis*	Proteobacteria Betaproteobacteria
Q82WU3	*Nitrosomonas europaea*	Proteobacteria Betaproteobacteria
Q9RAM7	*Methylobacillus flagellatus*	Proteobacteria Betaproteobacteria
Q5P233	*Azoarcus sp.*	Proteobacteria Betaproteobacteria
Q8Y0U6	*Ralstonia solanacearum*	Proteobacteria Betaproteobacteria
Q7NZ95	*Chromobacterium violaceum*	Proteobacteria Betaproteobacteria
P67042	*Brucella melitensis*	Proteobacteria Alphaproteobacteria
P67043	*Brucella suis*	Proteobacteria Alphaproteobacteria
Q89G35	*Bradyrhizobium japonicum*	Proteobacteria Alphaproteobacteria
Q98KH9	*Rhizobium loti*	Proteobacteria Alphaproteobacteria
Q6N447	*Rhodopseudomonas palustris*	Proteobacteria Alphaproteobacteria
Q92NQ5	*Rhizobium meliloti*	Proteobacteria Alphaproteobacteria
Q8UDS3	*Agrobacterium tumefaciens*	Proteobacteria Alphaproteobacteria
Q9A6H0	*Caulobacter crescentus*	Proteobacteria Alphaproteobacteria
Q6FZ91	*Bartonella quintana*	Proteobacteria Alphaproteobacteria
Q6G2S8	*Bartonella henselae*	Proteobacteria Alphaproteobacteria
Q5YPL7	*Nocardia farcinica*	Actinobacteria Actinobacteridae
Q6A761	*Propionibacterium acnes*	Actinobacteria Actinobacteridae
Q6AFI0	*Leifsonia xyli subsp. xyli*	Actinobacteria Actinobacteridae
Q8G3T9	*Bifidobacterium longum*	Actinobacteria Actinobacteridae
Q73VZ2	*Mycobacterium paratuberculosis*	Actinobacteria Actinobacteridae
Q9CBW0	*Mycobacterium leprae*	Actinobacteria Actinobacteridae
P67044	*Mycobacterium tuberculosis*	Actinobacteria Actinobacteridae
P67045	*Mycobacterium bovis*	Actinobacteria Actinobacteridae
Q6NIF2	*Corynebacterium diphtheriae*	Actinobacteria Actinobacteridae
Q8FR47	*Corynebacterium efficiens*	Actinobacteria Actinobacteridae
Q8NS38	*Corynebacterium glutamicum*	Actinobacteria Actinobacteridae

Q7MTB5	*Porphyromonas gingivalis*	Bacteroidetes	Bacteroidetes
Q8A639	*Bacteroides thetaiotaomicron*	Bacteroidetes	Bacteroidetes
Q64PV5	*Bacteroides fragilis*	Bacteroidetes	Bacteroidetes
D0JBG7	*Blattabacterium sp. subsp. Blattella germanica*	Bacteroidetes	Flavobacteria
D0J8U9	*Blattabacterium sp. subsp. Periplaneta americana*	Bacteroidetes	Flavobacteria
Q7UID0	*Rhodopirellula baltica*	Planctomycetes	Planctomycetacia
Q5WDS1	*Bacillus clausii*	Firmicutes	Bacillales
Q59212	*Bacillus licheniformis*	Firmicutes	Bacillales
Q65J44	*Bacillus licheniformis*	Firmicutes	Bacillales
Q9ANR7	*Bacillus mojavensis*	Firmicutes	Bacillales
P42326	*Bacillus subtilis*	Firmicutes	Bacillales
Q5D189	*Bacillus subtilis*	Firmicutes	Bacillales
Q9K7B5	*Bacillus halodurans*	Firmicutes	Bacillales
P54081	*Bacillus amyloliquefaciens*	Firmicutes	Bacillales
Q67JQ1	*Symbiobacterium thermophilum*	Firmicutes	Lactobacillales
Q5KZ25	*Geobacillus kaustophilus*	Firmicutes	Bacillales
Q97EV3	*Clostridium acetobutylicum*	Firmicutes	Clostridia
**Viruses**			
P07606	*Bacillus phage* phi3T	dsDNA viruses	Caudovirales
P00471	*Enterobacteria phage* T4	dsDNA viruses	Caudovirales
P90463	*Human herpesvirus* 8	dsDNA viruses	Herpesvirales
P06854	*Saimiriine herpesvirus* 2	dsDNA viruses	Herpesvirales
P12462	*Herpesvirus ateles*	dsDNA viruses	Herpesvirales
Q89940	*Equine herpesvirus* 2	dsDNA viruses	Herpesvirales
P09249	*Varicella-zoster* virus	dsDNA viruses	Herpesvirales
